# Do Sleep Disorders Positively Correlate with Dry Eye Syndrome? Results of National Claim Data

**DOI:** 10.3390/ijerph16050878

**Published:** 2019-03-11

**Authors:** Kyu-Tae Han, Ji Hyung Nam, Eun-Cheol Park

**Affiliations:** 1 Division of Cancer Control and Policy, National Cancer Center, Goyang 10408, Korea; kthan.phd@gmail.com; 2 Institute of Health Services Research, Yonsei University College of Medicine, Seoul 03722, Korea; 3 Department of Internal Medicine, Dongguk University Ilsan Hospital, Dongguk University College of Medicine, Goyang 10326, Korea; drnamesl@gmail.com; 4 Department of Medicine, Graduate School, Yonsei University, Seoul 03722, Korea; 5 Department of Preventive Medicine, Yonsei University College of Medicine, Seoul 03722, Korea

**Keywords:** sleep disorders, dry eye syndromes, quality of life, eye diseases

## Abstract

*Purpose*: Dry eye syndrome (DES) is a common disease with an increasing occurrence. Although DES symptoms are considered mild, it can reduce quality of life for individuals. Many studies on DES have been conducted, but these focused on the use of electronic devices. Here, we investigate an association between DES and sleep disorders in the context of emerging health issues. *Methods*: Our data came from the National Health Insurance Service (NHIS) National Sample Cohort, which included 44,366 patients and was based on a 1:1 matching method (sleep disorder patients vs. patients without sleep disorders) during 2012–2015. Using survival analysis with a Cox proportional hazard model, we identified an association of sleep disorders with DES. *Results*: About 16.7% of all patients were diagnosed with DES, and prevalence was higher in patients with sleep disorders (sleep disorders: 19.82%, no sleep disorders: 13.67%). Survival analysis showed that sleep disorders positively correlated with DES diagnosis (Hazard Ratio (HR): 1.320, 95% Confidence Interval (CI): 1.261–1.382, *p*-value < 0.0001). Positive trends were enhanced in males, younger patients, lower economic levels, and with higher severity of comorbid. *Conclusions*: Our findings suggest that sleep disorder was positively associated with DES. This correlation can be helpful in effective management of both sleep disorders and DES in South Koreans.

## 1. Introduction

South Korea has experienced many developments and changes in the last century, including new social and economic phenomena [1,2]. Overall socioeconomic levels and health status of South Koreans rapidly improved. However, with such rapid change, people face challenges, and most of them experience psychiatric problems caused by increasing stressful events [3]. Psychiatric problems can include mild stress as well as suicidal behavior, and sleep disorder is considered to be an important related factor. This is because sleep is substantial part of daily life, and deficit or excess of sleep can cause additional problems [4]. In addition, people without psychiatric problems could be faced with that [5,6].

According to the National Health Insurance Service (NHIS) in South Korea, the number of patients with organic sleep disorders is rapidly increasing each year (2010: 287,835 to 2016: 494,915) [7]. To solve this problem, patient and healthcare professional’s attentions have shifted to the importance of managing quality of sleep and problems related to sleep. Thus, previous studies have suggested that many problematic physical or psychiatric conditions were caused by sleep disorders, even if patients had no accompanying psychiatric problems [8,9,10]. Sleep quality and sleep duration are now known to be essential factors in managing health. Although many health problems are associated with sleep disorders, in this study, we focus on dry eye syndrome (DES).

DES is one of the most common eye diseases and according to NHIS reports, the number of cases is continuously increasing (2007: 1,426,549 to 2016: 2,244,627) [11]. Many studies have been conducted regarding this increase, and most healthcare professionals have suggested that increased electronic device usage, including smart phones, causes DES [12,13]. Although DES symptoms are considered mild, some psychiatric studies suggest that DES reduces the quality of an individual’s daily life due to uncomfortable stress [14]. In severe cases, DES can contribute to other physical symptoms, such as worsening vision. In addition, medical costs attributed to DES ($85.8 million in 2016, 10% increase per year) indicate that development of alternatives for effective management of DES is needed [11].

Even though some cross-sectional/case-control studies based on patient clinics have shown positive association between DES and sleep disorders [15,16,17], there are few large-scale or nationwide studies. In particular, no findings associate DES with sleep disorders using national claim data in South Korea, because DES (International Classification of Diseases [ICD]-10 code: H04.11; new classification in 2011) was not identified in healthcare claim data before 2011. Thus, in this study, we investigate the association between the organic sleep disorders and DES using national claim data in South Korea. Based on our results, we suggest helpful evidence for establishing alternatives or health policy to prevent new healthcare problems due to emerging health issues.

## 2. Methods

### 2.1. Study Population

The data used in this study was the NHIS National Sample Cohort 2002–2015, which was released by the NHIS in 2017 (research No. NHIS-2017-2-568). These data include 1,000,000 representative individuals who were randomly selected from the South Korean population in 2006. The data include all medical claims filed from January 2002 to December 2015. In consideration of the 2011 classification of DES in South Korean healthcare claim data, we first excluded all patients who were deceased, and then identified patients who were diagnosed with DES (ICD-10: H04.11) or sleep disorders (ICD-10: G47) before 2012, and assumed that these patients were newly diagnosed with DES or sleep disorders. We excluded patients who were diagnosed with DES before sleep disorders. Finally, we identified 22,183 patients with sleep disorders during 2012–2015. To design the matched cohort study using the sleep disorder patients as the case group, we established the control group by propensity score matching by adjusting for same sex, similar age (5-year intervals), and similar date of diagnosis for sleep disorders. We used a 1:1 matching method to select the control group (22,183 patients) who were not diagnosed with sleep disorders. Finally, the data used in this study contained the records of 44,366 patients from 2012 to 2015. This study was approved by an Institutional Review Board and the National Health Insurance Service Ilsan Hospital (NHIMC 2017-09-015).

### 2.2. Variables

The outcome variable in this study was whether patients were diagnosed with DES, according to the presence of sleep disorders. We identified the date of each patient’s first hospital visit with DES as the main diagnosis (either outpatient care or inpatient care, ICD-10 code H04.11) from 2012 to 2015 and categorized that patient into the DES diagnosis group.

The major variable of interest was the diagnosis of sleep disorders. In this study, sleep disorders were defined as organic sleep disorders (ICD-10: G47.x), not included non-organic sleep disorders (F41.x). Organic sleep disorder consists of insomnia, hypersomnia, disorders of the sleep-wake schedule, sleep apnea, narcolepsy/cataplexy, and other sleep disorders.

To investigate the association between DES and sleep disorders in this study, we included the following independent variables: sex, age, economic level, type of insurance coverage, Charlson comorbidity index (CCI), and region. Age was classified by 10 year intervals: less than 29 years, 30–39 years, 40–49 years, 50–59 years, 60–69 years, 70–79 years, and more than 80 years. Economic level was defined by insurance premiums in claim data. In South Korea, all people apply to the NHI and pay insurance premiums based on their economic level, determined by salary or property (generally about 7% of salary was paid as premium in 2017; this proportion changes every year). Thus, insurance premiums can indirectly reflect economic level. We classified economic level as: less than 30% (low income), 31–60%, 61–90%, and more than 91% (high income). Insurance coverage is divided into three types in South Korea. If patients are workers, employers in workplaces, public officials, private school employees, continuously insured persons, or daily paid workers at construction sites, they are defined as an NHI employee. Those with NHI employee insurance pay a regular portion of their average salary in contribution payments, and include spouses, dependents, siblings, and parents. The NHI self-employed insurance category includes people who do not fall into the NHI employee insurance group. Their contribution amount is set by taking into account their income, property, living standard, and rate of participation in economic activities. Medical aid beneficiaries are defined as patients with an income below the government-defined poverty level, or as those with a disability who were provided with free inpatient and outpatient care paid by government funds. Therefore, the type of insurance coverage represents each patient’s socioeconomic status. The CCI was calculated by weighing and scoring for comorbid conditions, with additional points added to account for comorbidities that may affect health outcomes of patients [18]. CCI was categorized into three groups: ‘0’, ‘1’, ‘2’, and ‘more than 3’.

### 2.3. Statistical Analysis

To investigate the association between DES and sleep disorders, we first examined the frequencies and percentages of the study population by DES diagnosis. Next, we performed a log-rank test and generated a Kaplan-Meier survival curve using product-limit method to compare the rates of diagnosis for DES between patients with or without sleep disorders. Finally, we performed survival analysis using the Cox proportional hazard model to identify the association of sleep disorder with DES, adjusting for independent variables. In addition, to examine the differences in the association between DES and sleep disorder, we performed sub-group analysis for survival analysis according to sex, age, economic level, and CCI. All statistical analyses were performed using SAS statistical software version 9.2 (SAS Inc., Cary, NC, USA).

## 3. Results

We analyzed 44,366 patients diagnosed with sleep disorders or not during 2012–2015, using a 1:1 propensity score matching method (sleep disorder patients vs. patients without sleep disorders). Table 1 shows the distribution of study population by each variable. About 16.7% of total patients were diagnosed with DES, and its prevalence was higher in patients with sleep disorders (sleep disorders: 19.82%, no sleep disorders: 13.67%). By sex, female patients had more frequent DES diagnosis than males. Older patients were more often diagnosed with DES than younger patients, and patients with higher economic levels were more diagnosed with DES than low income groups. These trends were also observed when results were analyzed by the types of insurance coverage: NHI employee insured patients were more diagnosed with DES than Medical Aid patients. As an indication of health severity for each patient, the CCI had inverse trends with DES diagnosis. In addition, patients who lived in metropolitan areas visited medical institutions due to DES more often than rural patients.

**Figure 1:**
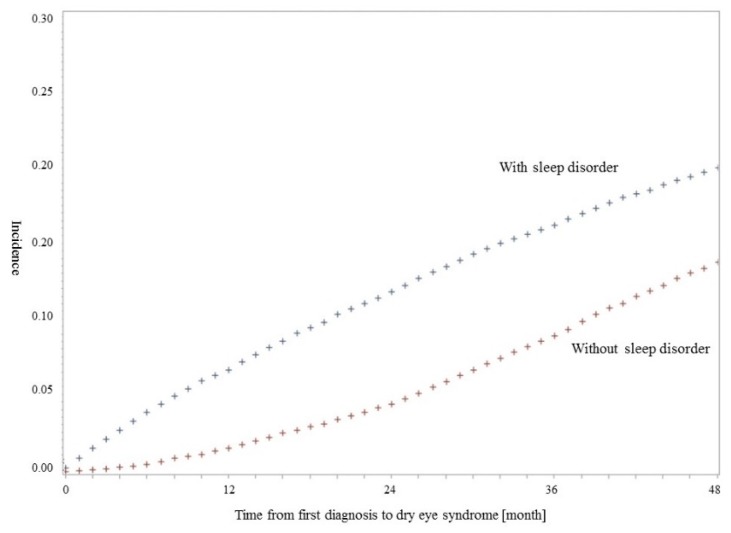
Kaplan-Meier survival curve for incidence of dry eye syndrome (DES). The results of log-rank test for time to DES by diagnosis of sleep disorders were statistically significant.

Figure 1 shows the Kaplan-Meier survival curve generated using product-limit methods to compare the incidence of DES during the study period between patients with sleep disorders and without sleep disorders. In the survival curve, patients with sleep disorders were more often diagnosed with DES than patients without sleep disorders (*p*-value for log-rank test < 0.0001).

Table 2 shows the results of survival analysis using the Cox proportional hazard model to identify an association between DES and sleep disorder while adjusting for other independent variables. Sleep disorder positively correlated with DES diagnosis (HR: 1.320, 95% CI: 1.261–1.382, *p*-value < 0.0001). Of the independent variables, female and older patients were more associated with DES than other variables. The top 10% of economic levels and patients insured as NHI employees were more often diagnosed with DES than lower socioeconomic levels. In contrast, the results comparing CCI with DES were not statistically significant. Regional variables were also associated with DES (Metropolitan = HR: 1.080, 95% CI: 1.039–1.122, *p*-value < 0.0001).

**Figure 2:**
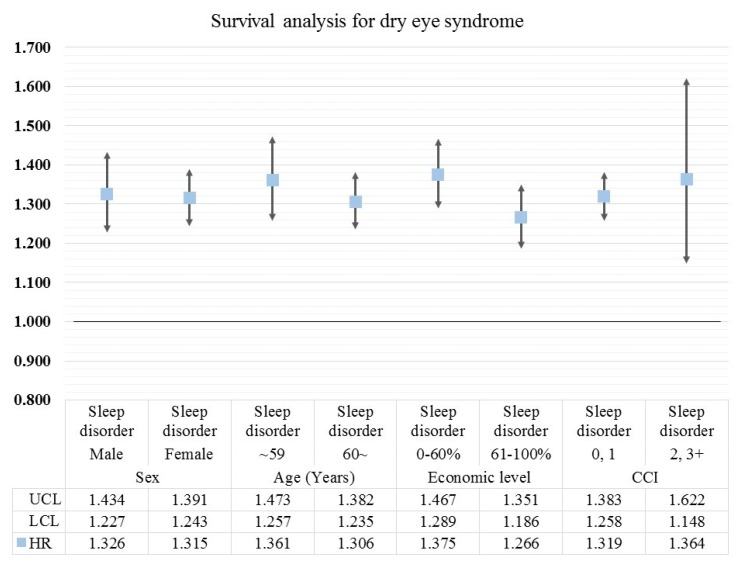
The results of sub-group analysis for survival analysis according to sex, age, economic level, and CCI. The HR as marked by the square points was calculated by survival analysis to investigate the association between sleep disorder and DES. Results were considered statistically significant if each bar marked to SD did not reach the cut-off line of 1.000.

Figure 2 shows the results of sub-group analysis for survival analysis. When organizing the results by sex, sleep disorder was more associated with DES in male patients (male, sleep disorders = HR: 1.326, 95% CI: 1.227–1.434, *p*-value < 0.0001; female, sleep disorders = HR: 1.315, 95% CI: 1.243–1.391, *p*-value < 0.0001). Positive trends were increased in younger patients than in older patients. In addition, patients at low economic levels had more positive correlation between DES and sleep disorders than higher income groups. On the other hand, the increased trends by sleep disorders were higher in patients with a higher CCI value than in patients with a low CCI (0/1, sleep disorders = HR: 1.319, 95% CI: 1.258–1.383, *p*-value < 0.0001; 2/3+, sleep disorders = HR: 1.364, 95% CI: 1.148–1.622, *p*-value = 0.0004).

## 4. Discussion

Considering emerging trends in healthcare, there is a continuous need for alternative strategies for effective management. For most health issues, problems include a lack of optimal alternatives. In some parts of the South Korean healthcare arena and due to a challenging social atmosphere, there are still uncontrollable healthcare issues that contribute to psychiatric problems, including stress and suicide. Sleep is one such health problem. In recent times, most South Koreans have had hectic days and naturally, the duration of sleep was less than other countries [19]. Thus, many studies investigate the deficits in sleep quality and duration in South Korea, and the major focus of these findings has been on severe symptoms, such as cancer, cardiovascular disease, and psychiatric symptoms. However, although DES cases have continuously increased each year, there are few studies about risk factors for DES or its association with sleep disorders. Therefore, we investigated the association between these conditions, considering that cases of both diseases are increasing.

Our findings suggest that the sleep disorders, which were not caused by psychiatric problems, was positively associated with increasing DES in South Korea. In results of previous studies, the association between sleep disorders and DES was analyzed; in particular, shorter sleep duration was a major contributor to DES [16,20,21]. In addition, some professionals suggest that the DES could be caused by reducing quality of sleep, such as by sleep disturbance [17]. A recent study also showed that sleep disorders were prevalent in DES, and sleep quality was affected by symptom severity in DES patients [22]. However, due to the lack of data to study such an association because of the new classification of DES in claim data, there are no clear findings associating DES and sleep disorders in South Korea, except in the context of lacrimal system disorder (ICD-10: H04) or a cross-sectional study [23]. In a randomized clinical trial study, it was suggested that sleep deprivation reduces tear secretion and could trigger ocular surface diseases [24]. However, DES has an unknown etiology and a mechanism to support the association of sleep disorders with DES is still unclear [25]. As included various sleep disorders such as hypersomnia and disturbed sleep time as well as insomnia, our results could not provide an evidence supporting mechanisms relating to sleep deprivation. Our findings about the emerging diseases of sleep disorder and DES may inform decisions made by healthcare professionals. Future researches are warranted to logically support these associations.

Our study also has some interesting findings based on sub-group analysis. Generally, people with more accessibility or attention for healthcare, such as high income or metropolitan residents, was more diagnosed with DES. However, the positive correlation between DES and sleep disorders was greater in male, younger, and poorer groups. This was because these associations were more likely to affect DES risk in populations with lower initial risk. In other words, in populations with low risk for DES, sleep disorder could be a major risk factor. In developing effective approaches, these findings provide information for managing DES. On the other hand, patients with more health severity as determined by CCI had a more positive correlation between DES and sleep disorder. This observation might be explained by the clinical vulnerability of this population. Thus, in severe health patients, managing sleep conditions could be a key factor in preventing DES.

Our study has several strengths related to the study population and methodology. First, to our best knowledges, this study is the first attempt to investigate an association between sleep disorder without psychiatric problems and DES in South Korea. As previously mentioned, DES was clear classified as distinct from lacrimal system disorder after 2011, and the cohort data which included this information was only recently disclosed. Thus, there are few studies about factors associated with DES in South Korea. Therefore, this study is helpful in informing clinical decisions or evidence-based health policy for managing new emerging diseases. Second, the South Korean government has introduced the NHI for the overall population and collects healthcare claims information related to the payment system. The data used in this study was from a retrospective cohort study, which included healthcare claim data from more than 10 years of the NHI system. Thus, the results generated from this data provide more detailed analysis than other type of data. Third, in this study, we used a matched cohort design, using propensity score matching (1:1). Therefore, our findings minimize selection bias and recall bias [26]. Finally, previous studies showed that pre-existing clinical conditions could adversely affect patient outcomes. Therefore, we adjusted for previous psychiatric hospitalization and CCI in this study.

Our study also has some limitations. First, because we use insurance claim data, we depend on the ICD-10 code to define the diagnoses of sleep disorder and DES. Therefore, there could be an under- or overestimation of diagnosis in the study population. Nevertheless, the data included the overall population in South Korea and is only limited based on using claim data. Second, the severity of sleep disorder could be influenced by using pharmaceuticals. However, psychotic drugs for sleep disorder (G47), such as Benzodiazepines, are not covered by NHI [27]. Thus, we could not measure medicine as a severity factors for sleep disorder due to the nature of the claim data. Furthermore, we could not evaluate the effect of the drug treatment of sleep disorders on DES. Third, previous studies identified other factors that could affect the patient’s condition, such as use of electronic devices, job status, and social relationship. However, we could not consider such factors in this study due to the limitations of the healthcare claim data. Fourth, our claim data was not available to classify DES based on the subtypes such as evaporative or secretive type. It would be interesting to evaluate what kind of DES better correlates with sleep disorders in the future. Fifth, the positive association between sleep disorders and DES may be linked to the socioeconomic status of South Korea. Thus, it is unreasonable to generalize our findings to other countries with different socioeconomic and demographic characteristics.

Although there are some limitations related to the methods of this study, our findings suggest that sleep disorders could be associated with incidence of DES, and that this association was greater in patients with less access to medical resources and with higher severity of comorbid clinical conditions. Our findings suggest that there needs to be more effective alternatives for managing emerging diseases, such as sleep disorder or DES. A solution for these issues will improve the quality of life for South Koreans.

## 5. Conclusions

Our findings suggest that sleep disorders could cause DES. This relationship can be helpful in effective management of both sleep disorders and DES in South Koreans. In addition, strategies need to be developed for managing emerging health problems, including sleep disorders or DES.

## Figures and Tables

**Table 1:** General characteristics of the study population.

Variables	Total		Dry Eye Syndrome			
			Diagnosed		None	
	*N*	%	*N*	%	*N*	%
**Sleep disorder**						
Yes	22,183	50.00	4397	19.82	17,786	80.18
No	22,183	50.00	3032	13.67	19,151	86.33
**Sex**						
Male	19,893	44.84	2564	12.89	17,329	87.11
Female	24,473	55.16	4865	19.88	19,608	80.12
**Age (Years)**						
<29	1312	2.96	125	9.53	1187	90.47
30–39	3498	7.88	515	14.72	2983	85.28
40–49	5694	12.83	736	12.93	4958	87.07
50–59	7804	17.59	1145	14.67	6659	85.33
60–69	10,385	23.41	1902	18.31	8483	81.69
70–79	7227	16.29	1466	20.29	5761	79.71
>80	8446	19.04	1540	18.23	6906	81.77
**Economic level**						
<30% (low)	12,195	27.49	2007	16.46	10,188	83.54
31–60%	10,805	24.35	1659	15.35	9146	84.65
61–90%	14,577	32.86	2504	17.18	12,073	82.82
>91% (high)	6789	15.30	1259	18.54	5530	81.46
**Types of insurance coverage**						
Medical aid	1984	4.47	307	15.47	1677	84.53
NHI, self–employed insured	14,581	32.87	2362	16.20	12,219	83.80
NHI, employee insured	27,801	62.66	4760	17.12	23,041	82.88
**Charlson comorbidity index**						
0	37,081	83.58	6226	16.79	30,855	83.21
1	4682	10.55	802	17.13	3880	82.87
2	1771	3.99	280	15.81	1491	84.19
3+	832	1.88	121	14.54	711	85.46
**Region**						
Metropolitan	20,021	45.13	3496	17.46	16,525	82.54
Others	24,345	54.87	3933	16.16	20,412	83.84
**Total**	44,366	100.00	7429	16.74	36,937	83.26

NHI: National Health Insurance.

**Table 2:** Results of survival analysis using Cox proportional hazard model for association between sleep disorders and dry eye syndrome.

Variables	Dry Eye Syndrome			
	HR	95% CI		*p*-Value
		Lower	Upper	
**Sleep disorder**				
Yes	1.320	1.261	1.382	<0.0001
No	1.000	–	–	–
**Sex**				
Male	0.640	0.615	0.667	<0.0001
Female	1.000	–	–	–
**Age (Years)**				
<29	1.000	–	–	–
30–39	1.594	1.350	1.882	<0.0001
40–49	1.473	1.255	1.729	<0.0001
50–59	1.688	1.445	1.973	<0.0001
60–69	2.123	1.822	2.475	<0.0001
70–79	2.479	2.124	2.894	<0.0001
>80	2.225	1.906	2.597	<0.0001
**Economic level**				
<30% (low)	1.000	–	–	–
31–60%	0.960	0.907	1.016	0.1622
61–90%	1.029	0.977	1.084	0.2844
>91% (high)	1.086	1.020	1.155	0.0094
**Types of insurance coverage**				
Medical aid	0.949	0.858	1.049	0.3056
NHI, self–employed insured	0.940	0.902	0.980	0.0037
NHI, employee insured	1.000	–	–	–
**Charlson comorbidity index**				
0	1.000	–	–	–
1	1.053	0.991	1.119	0.0931
2	1.019	0.928	1.119	0.6983
3+	1.012	0.882	1.161	0.8621
**Region**				
Metropolitan	1.080	1.039	1.122	<0.0001
Others	1.000	–	–	–
